# Cardiovascular risk factor control in British adults with diabetes mellitus: Retrospective cohort study

**DOI:** 10.1002/edm2.114

**Published:** 2020-01-27

**Authors:** Sophie É. Collins, Brendan Cord Lethebe, Tyler Williamson, Finlay A. McAlister

**Affiliations:** ^1^ Division of General Internal Medicine Faculty of Medicine & Dentistry University of Alberta Edmonton AB Canada; ^2^ Faculty of Rehabilitation Medicine University of Alberta Edmonton AB Canada; ^3^ Clinical Research Unit Cumming School of Medicine University of Calgary Calgary AB Canada

**Keywords:** cardiovascular risk factors, diabetes mellitus, targets, treatment

## Abstract

Using primary care electronic medical records (the United Kingdom Health Improvement Network Database 2003‐2015), we examined the control of cardiovascular risk factors in the first year after diagnosis in British adults with diabetes mellitus. Among 292 170 individuals with diabetes receiving frequent outpatient management (median of 16 primary care visits in the prior year), control of cardiovascular risk factors a median of 354 days after diagnosis was suboptimal: 14.7% had HbA1C < 7%, SBP < 140 mm Hg, LDL cholesterol ≤1.8 mmol/L or taking a statin, and were nonsmokers (the proportion dropped to 7.5% if the SBP target was defined as <130 mm Hg). While 90.4% had an LDL cholesterol ≤1.8 mmol/L or were taking a statin, and 86.0% were nonsmokers, only 52.0% had HbA1C < 7% and 53.1% had SBP < 140 mm Hg (29.8% had SBP < 130 mm Hg) despite 71.4% taking antihypertensive agents. Thus, there is still a need for quality improvement strategies that target all atherosclerotic risk factors in individuals with diabetes and not just glycaemic control.

## Graphical abstract

Using primary care electronic medical records (the United Kingdom Health Improvement Network Database 2003‐2015), we examined the control of cardiovascular risk factors in the first year after diagnosis in British adults with diabetes mellitus. Among 292,170 individuals with diabetes receiving frequent outpatient management (median of 16 primary care visits in the prior year), control of cardiovascular risk factors a median of 354 days after diagnosis was suboptimal: 14.7% had HbA1C < 7%, SBP < 140 mm Hg, LDL cholesterol <1.8 mmol/L or taking a statin, and were nonsmokers (the proportion dropped to 7.5% if the SBP target was defined as <130 mm Hg). Thus, there is still a need for quality improvement strategies that target all atherosclerotic risk factors in individuals with diabetes and not just glycaemic control.

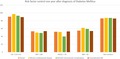

## 1 INTRODUCTION

Although control of multiple cardiovascular (CV) risk factors leads to substantial reduction in risk of cardiovascular events and death in patients with type 2 diabetes,^1^ recent reports from the TECOS^2^ and BARI‐2D^3^ trials suggested that only one third of individuals with type 2 diabetes in those trials exhibited optimal control of their other cardiovascular risk factors. This mirrors findings from the National Health and Nutrition Examination Surveys in the United States.^4^ However, participants in randomized trials and cohort studies are often healthier and more adherent with lifestyle modifications than nonparticipants, and whether control rates are better or worse in real‐world practice is unknown. Therefore, the purpose of this study is to examine control of all cardiovascular risk factors in adults with newly diagnosed diabetes cared for by UK primary care physicians, and to explore whether control patterns varied by comorbidity profiles. We used guideline recommendations on cardiovascular risk factor management in individuals with diabetes (https://www.diabetes.co.uk/diabetes-health-guidelines.html last accessed 1 August 2019) to define optimal treatment goals.

## 2 METHODS

### 2.1 Cohort selection

As described in detail elsewhere,^5^ we used de‐identified data from primary care electronic medical records (the United Kingdom Health Improvement Network [THIN] Database) to examine risk factor control in patients with diabetes mellitus aged 20 years or older at the time of diagnosis. We used read clinical encounter codes (entered by the clinician caring for the patient) and free word searching in the ontology navigator for any glucose‐lowering drug prescriptions to identify patients with a new diagnosis of diabetes. In the twelve years (2003‐2015) we examined, 670 National Health Service (NHS) primary care practices contributed data from more than 14 million patients to the THIN, 4.4 million of whom were followed longitudinally. While specialty clinics are not included in the THIN Database, any specialist recommendations to the primary care physician are captured. The THIN Data set is representative of the UK population, and the diagnostic coding accuracy is high for chronic conditions such as diabetes.^6^

Our cohort consists of patients with newly diagnosed diabetes seen between 2003 and 2015 who had a recorded measurement of their HbA1C and systolic blood pressure (SBP) at least 6 months after they were diagnosed with diabetes but before one year. We defined the index date for assessing CV risk factor control as the time of their first HbA1C done at least 6 months after diagnosis.

### 2.2 Definition of risk factor control

We defined CV risk factor control for each patient on the basis of laboratory results and physical measures recorded at least 6 months after the initial diagnosis of diabetes (in order to give physicians and patients time to implement any changes) but before one year. In the case of multiple measurements, we used those closest to the time of the index HbA1C measurement.

### 2.3 Covariates

The specific variables included are detailed in the Table 1 and were based on diagnoses assigned by their primary care physician.

**Table 1:** Patient characteristics, healthcare utilization, laboratory results and prescribed drugs within 12 mo after diagnosis of diabetes mellitus

Characteristics	Overall (n = 292 170)	Patients with cardiovascular disease (IHD, HF, or cerebrovascular disease) (n = 26 215)	Patients with CKD (n = 3166)	Patients with diabetic complications (retino, neuro or nephropathy) (n = 16 396)	Patients with uncomplicated diabetes mellitus (n = 249 381)
Age, mean (SD)	61.7 (15.6)	69.6 (11.2)	65.7 (14.4)	61.7 (14.6)	60.8 (15.8)
Female, % (n)	45.2 (132 167)	39.5 (10 358)	36.4 (1151)	42.1 (6897)	46.1 (114 862)
Number of primary care physician visits in the year prior to risk factor measurement, mean (SD)	16.3 (12.4)	20.5 (15)	21.3 (15.3)	17.9 (13)	15.7 (12)
Charlson Score, mean (SD)	1.1 (0.9)	1.8 (1.2)	2.3 (1.5)	2.4 (1.2)	1 (0.8)
Specific Comorbidities (not mutually exclusive)					
Hypertension	15.6 (45 470)	21.2 (5563)	25 (790)	19.4 (3180)	14.7 (36 767)
Chronic kidney disease	1.1 (3166)	2.4 (633)	100 (3166)	4.1 (668)	0 (0)
Ischaemic heart disease (including prior myocardial infarction or CABG)	6 (17 501)	66.8 (17 501)	12.1 (384)	7.3 (1197)	0 (0)
Heart failure	1.8 (5256)	20 (5256)	8 (253)	2.6 (431)	0 (0)
Cerebrovascular disease	2.1 (6133)	23.4 (6133)	4.1 (130)	2.6 (425)	0 (0)
Chronic obstructive pulmonary disease	3.3 (9523)	6 (1585)	5.3 (167)	3.1 (506)	3 (7444)
Cancer	0.1 (347)	0.2 (44)	0.4 (12)	0.1 (11)	0.1 (285)
Depression	4.6 (13 484)	5.4 (1425)	5 (158)	4.9 (806)	4.5 (11 274)
Dementia	0.6 (1818)	1.3 (328)	0.8 (26)	0.6 (92)	0.6 (1402)
Diabetes complications					
‐retinopathy	5.2 (15 131)	6.2 (1633)	11.3 (359)	92.3 (15 131)	0 (0)
‐neuropathy	0.4 (1031)	0.6 (169)	1.1 (34)	6.3 (1031)	0 (0)
‐nephropathy	0.2 (566)	0.4 (112)	13.4 (423)	3.5 (566)	0 (0)
‐any of the above	5.6 (16 396)	7 (1834)	21.1 (668)	100 (16 396)	0 (0)
Risk Factor control (based on measurements closest to index HbA1C done at least 6 mo after, but within 12 mo of, diabetes diagnosis)					
SBP < 140 mm Hg	53.1 (155 028)	49.9 (13 080)	45 (1426)	48.9 (8022)	53.6 (133 792)
SBP < 130 mm Hg	29.8 (87 041)	28.7 (7533)	25.4 (804)	27 (4423)	30.1 (75 030)
BMI* < 25	21.9 (4983)	26.6 (543)	26.4 (66)	22.6 (257)	21.3 (4170)
BMI* < 30	54.9 (12 508)	61.9 (1262)	60 (150)	59.2 (672)	53.9 (10 560)
LDL cholesterol ≤ 1.8 mmol/L OR taking statin**	90.4 (153 052)	97 (18 906)	96.2 (2123)	92.9 (8874)	89.3 (125 229)
HbA1c < 7%	52 (151 864)	51.4 (13 473)	48.2 (1525)	39.9 (6545)	52.7 (131 505)
Smoking Status***					
Current	14 (40 818)	12.6 (3311)	13.8 (438)	12.8 (2102)	14.2 (35 351)
Never	39.3 (114 808)	31.6 (8271)	35.3 (1119)	39.6 (6499)	40.1 (99 922)
Former	46.1 (134 835)	55 (14 429)	50.3 (1592)	47.1 (7725)	45.2 (112 657)
Summary of Cardiovascular Risk Factor Control****					
SBP < 140 mm Hg AND HbA1c < 7% AND (LDL cholesterol ≤ 1.8 mmol/L or taking statin) AND Nonsmoker	14.7 (39 258/267 516)	18.5 (4641/25 123)	14.3 (430/3000)	11.1 (1726/15 490)	14.5 (32 821/226 784)
SBP < 130 mm Hg AND HbA1c < 7% AND (LDL cholesterol ≤ 1.8 mmol/L or taking statin) AND Nonsmoker	7.5 (20 961/278 179)	10.5 (2704/25 635)	8.2 (253/3073)	5.8 (917/15 926)	7.3 (17 316/236 476)
Drugs prescribed within 120 d preceding the index HbA1C measurement (not mutually exclusive)					
Insulin	17 (49 623)	18.6 (4867)	29.6 (936)	34 (5570)	15.8 (39 454)
Any oral glucose‐lowering agent	58.2 (170 129)	62.1 (16 284)	60.2 (1906)	58.9 (9661)	57.8 (144 092)
ACE inhibitor or ARB	46.8 (136 812)	68.6 (17 982)	75.9 (2402)	54.4 (8922)	44 (109 808)
Statin	51.9 (151 782)	71.8 (18 831)	66.7 (2112)	53.8 (8821)	49.8 (124 090)
Any Antihypertensive Agent(s)	71.4 (208 666)	88 (23 064)	84.5 (2675)	71.1 (11 654)	69.7 (173 776)
Antiplatelet agent	37.9 (110 589)	81.9 (21 481)	56.6 (1791)	42.8 (7012)	33.1 (82 566)

Note

Patient characteristics are reported as proportions for categorical variables (with numbers in brackets). All *P* values across rows <0.001.

Abbreviations: ACE, angiotensin converting enzyme; ARB, angiotensin receptor blocker; BMI, body mass index.

* BMI only available for 22 786 patients.

** LDL values or information on statin prescribing missing for 122 865 patients.

*** Smoking status missing for 1709 patients.

**** Denominators for summary risk factor control represent the number of patients with all included variables available.

### 2.4 Statistical analysis

Patient characteristics were reported as means and standard deviations for continuous variables (and compared using t tests and one‐way ANOVA), and categorical variables were reported as proportions (and compared using chi‐squared tests).

### 2.5 Ethics

We were granted a waiver of informed consent by the University of Calgary Health Research Ethics Board (REB15‐0203_REN3) because we used de‐identified data from the THIN database obtained by the Cumming School of Medicine at the University of Calgary under license from IQVIA (IMS Quintiles VIA—see http://www.iqvia.com).

## 3 RESULTS

Of 406 649 individuals with diabetes, 292 170 (mean age 61.7 years) had both HbA1C and SBP measured 6‐12 months after diabetes diagnosis and formed the sample for this study. The median time from diabetes diagnosis to the risk factor assessments we examined was 354 days, and the median number of primary care physician visits in the year prior to the assessment of risk factor control was 16 (Table 1.). At the time of risk factor assessment, the mean HbA1C was 7.4% (52.0% had HbA1C < 7%), mean SBP was 138.2 mm Hg (53.1% had SBP < 140 mm Hg and 29.8% had SBP < 130 mm Hg), 71.4% were taking antihypertensive agents, 90.4% of patients had an LDL cholesterol ≤1.8 mmol/L or were taking a statin, and 86.0% of patients were current nonsmokers—Table 1. Control of glycaemia, BP and cholesterol was significantly better in patients with uncomplicated diabetes than in those with concomitant cardiovascular disease, CKD or diabetic microvascular complications (all *P* < .001). However, 14.7% of our cohort had HbA1C < 7%, SBP < 140 mm Hg, LDL cholesterol ≤1.8 mmol/L or were taking a statin, and were nonsmokers (the proportion dropped to 7.5% if the SBP target was <130 mm Hg)—Table 1.

## 4 DISCUSSION

We found that only one seventh of patients with type 2 diabetes receiving close follow‐up with UK primary care physicians had optimal risk factor profiles approximately one year after diagnosis of their diabetes. While control of lipids and nonsmoking rates were reasonably high, the frequency of SBP control was low and poorer than glycaemic control despite nearly three quarters of patients taking antihypertensive therapy. This is an important gap since SBP is the strongest driver of cardiovascular outcomes in diabetes (with quadruple the attributable risk for mortality and triple the attributable risk for cardiovascular events as hyperglycaemia in the Framingham study),^7^ the benefits of lowering blood pressure^8^ surpass those of lowering glucose in individuals with diabetes mellitus,^9^ and antihypertensives are the most cost‐effective cardiovascular prevention therapies in type 2 diabetes.^10^

The suboptimal control of cardiovascular risk factors in patients with type 2 diabetes we found in UK primary care practices is actually better than those reported in the United States and European studies.^4^, ^11^, ^12^, ^13^, ^14^ For example, a recent publication from the Swedish National Diabetes Register reported that only 5% of adults with type 2 diabetes were nonsmokers, did not have albuminuria, had BP < 140/80 mm Hg, LDL cholesterol <2.5 mmol/L and HbA1C < 7.0.^13^ Importantly, as the number of uncontrolled risk factors increased so did the risk of subsequent cardiovascular events.^13^ However, there is clearly still room for improvement and a recent systematic review of 42 randomized trials on improving management of type 2 diabetes documented that most primary care–based interventions focused on glycaemic management rather than total CV risk.^15^ However, there is a rich vein of literature on the efficacy of chronic disease management programs run by other healthcare professionals in collaboration with primary care physicians for optimizing total CV risk factor profiles in individuals with diabetes.^16^, ^17^

### 4.1 Limitations

Despite the availability of detailed clinical data in a large population‐based sample of adults with a new diagnosis of diabetes, there are some limitations to our study that should be acknowledged. First, the primary care clinical records may have under‐reported some comorbidities (particularly likely for conditions like dementia or depression). Additionally, we did not have access to data related to other factors that have the potential to influence clinical decision making such as patient socioeconomic status, patient values and preferences, specialist involvement in patient care, or local resource availability. Because of this, we chose not to perform multivariate analyses to explore whether specific comorbidities or patient factors were associated with CV risk factor control to avoid potentially misleading conclusions. Third, although we focused on only one set of measurements approximately one year after diagnosis of diabetes and did not examine any changes over time, we previously reported in this cohort that the HbA1C or SBP values changed little when re‐measured later.^5^ Finally, we had to exclude 114 479 patients with type 2 diabetes in the THIN database as they did not have data on their SBP or HbA1C in the first year after diagnosis of their diabetes.

## 5 CONCLUSION

In conclusion, nearly half of adults in our cohort newly diagnosed with diabetes mellitus exhibited suboptimal control of glucose (HbA1C > 7%) or SBP (>140 mm Hg), and over 85% exhibited suboptimal control of at least one cardiovascular risk factor despite frequent primary care visits. Despite a recent flurry of literature suggesting that individuals with diabetes may be over‐treated, our study highlights the continued need for primary care quality improvement strategies in type 2 diabetes that focus on all atherosclerotic risks and not just glycaemic control.

## CONFLICTS OF INTEREST

All authors have completed the ICMJE uniform disclosure form at http://www.icmje.org/coi_disclosure.pdf and declare: no support from any organization for the submitted work; no financial relationships with any organizations that might have an interest in the submitted work in the previous three years; and no other relationships or activities that could appear to have influenced the submitted work.

## AUTHORS' CONTRIBUTIONS

FM involved in study conception and design; BL and TW involved in data attainment and analysis; all authors involved in interpretation of data and subsequent revisions; SC and FM contributed to first draft of manuscript.

## Data Availability

The data that support the findings of this study are available under license from IQVIA (IMS Quintiles VIA—see http://www.iqvia.com) but restrictions apply to the availability of these data, which were used under license for the current study, and so are not publicly available.
